# The relationship between self-compassion and teacher self-efficacy in early childhood teachers: the mediating role of emotional exhaustion and the moderating role of core self-evaluation

**DOI:** 10.3389/fpsyg.2026.1828901

**Published:** 2026-06-18

**Authors:** Yuping Li, Dongqun Zhao

**Affiliations:** 1 School of Nursing and Health Care, Luoyang Polytechnic, Luoyang, Henan, China; 2 School of Preschool Education, Luoyang Normal University, Luoyang, Henan, China

**Keywords:** core self-evaluation, early childhood teachers, emotional exhaustion, self-compassion, self-efficacy

## Abstract

**Background:**

The professional psychological state of early childhood teachers is associated with their teaching quality and career stability. As a core professional psychological trait, self-efficacy is related to various psychological factors in its formation and stability. To better understand the underlying mechanism, this study adopts the Conservation of Resources Theory. We propose a moderated mediation model. This model examines the mediating role of emotional exhaustion in the association of self?compassion with self?efficacy in early childhood teachers. Whether core self-evaluation plays a moderating role is also examined.

**Methods:**

Standardized measures of self-compassion, emotional exhaustion, core self-evaluation, and self-efficacy are used in this work. We collected data from 552 Chinese early childhood educators. We used the PROCESS Macro 4.2 plugin in SPSS to perform path analysis.

**Results:**

Self-compassion showed a strong positive correlation with self-efficacy. Emotional exhaustion showed a partial mediating role in this relationship. The completely standardized indirect effect was 0.135 (95% bootstrap CI [0.095, 0.175]). Core self-evaluation positively moderated emotional exhaustion and self-efficacy.

**Conclusions:**

This study takes an empirical perspective on the correlates of self?efficacy and describes a theoretical basis for intervention design. These interventions are intended to be associated with the professional mental health of these teachers.

## 1 Introduction

To pursue high-quality development in early childhood education, the overall health and sense of self-efficacy of early childhood teachers have become increasingly important. These factors provide the foundation for quality early childhood education and ultimately support each child's healthy growth (Pan et al., 2022). The work of an early childhood teacher essentially requires continuous emotional investment. Teachers must constantly engage in high-intensity emotional labor and effectively manage complex role expectations. This is indispensable for completing daily tasks, including looking after children, conducting teaching interactions, and maintaining collaboration with families (Goldstein, 2007). According to the perspective of the Conservation of Resources (COR) theory, when people are under stress, they will consciously protect their limited psychological resources (Hobfoll, 1989). Significant resource loss is closely related to an individual's job adaptation and core self-evaluation (Yang et al., 2025). Therefore, identifying and cultivating the positive personal traits that are associated with early childhood teachers maintain their psychological resources is of significant practical importance. Self-compassion means treating oneself with kindness and tolerance, and accepting one's own limitations. This personal trait can become a protective resource for early childhood teachers, helping them cope with the stress in their work (Neff, 2003). Generally speaking, educators who possess a strong ability for self-care can more effectively regulate their emotions and maintain a positive mental state. However, the way self-compassion and self-efficacy are linked remains unclear and requires further investigation. According to the COR theory, self-compassion can be understood as a resource-generating personal trait. It is associated with the prevention of a downward loss spiral by reducing self-criticism and emotional exhaustion, and it is linked to conditions for an upward gain spiral by replenishing psychological resources like resilience and self-efficacy. Thus, self-compassion may correspond to both the interruption of resource loss and the presence of resource gain in early childhood teachers' occupational wellbeing (Hobfoll, 2001). Emotional exhaustion is the core indicator of psychological resource depletion. This state may function as a mediator linking self-compassion to self-efficacy (Chen et al., 2023; Zhang et al., 2022). Self-compassion may be associated with lower levels of emotional exhaustion by promoting adaptive emotion regulation and reducing self-criticism (Moè and Katz, 2020). Greater emotional exhaustion are often coincides with the depletion of the psychological resources needed to maintain self-efficacy (Yan et al., 2025). It is worth noting that different early childhood teachers may exhibit differences in this psychological process. Core self-evaluation is a fundamental and stable assessment of one's own abilities and values. It is usually associated with more effective strategies for resource protection. Core self-evaluation is also associated with maintaining a more positive self-perception and coping beliefs when they are experiencing emotional resource drain (Guo et al., 2019; Judge et al., 1997). From this perspective, core self-evaluation may act as a key moderator in the association of emotional exhaustion with self-efficacy.

The current research has explored the pairwise correlations among these variables. Research on integrating these variables into a unified model is still relatively limited. In the research, how emotional exhaustion mediates and core self-evaluation moderates within this integrated model warrants systematic examination. Therefore, this study probes early childhood teachers via COR theory, focusing specifically on the association linking self-compassion to self-efficacy among them. The work builds a moderated mediation model to deepen understanding of the processes associated with early childhood self-efficacy. This work provides a theoretical underpinning that is related to teachers' career psychological health and early childhood education quality.

## 2 Hypotheses

### 2.1 Self-compassion and self-efficacy

Self-compassion refers to the ability of an individual to respond to personal failures or troubles by making a kind self-judgment. It enables individuals to accept their own pain, reserve a certain space for it, and also view it as an integral part of themselves (Neff, 2003). It comprises three core dimensions: self-kindness, mindfulness, and common humanity (Neff, 2009). For early childhood teachers, self-compassion often manifests itself in actively reducing self-criticism when facing professional challenges. These challenges include managing the behavior of young children, designing teaching plans, and establishing effective communication with families. This means that teachers must recognize the limitations of their teaching and maintain an open mindset, reflecting and adjusting continuously (O'Hara-Gregan, 2023). Early childhood teachers engage in more frequent emotional labor and have more complex interpersonal interactions than teachers at other educational stages. These interactions involve young children's care requirements as well as the need for timely communication with their parents. These professional features make them more prone to self-doubt and emotional exhaustion, so self-compassion is particularly important for this group. In emotional regulation and resource conservation, self-compassion plays a crucial role (Sak et al., 2025).

Self-compassion, a positive regulatory resource, correlates theoretically with self-efficacy (Liao et al., 2021). Self-efficacy is defined as a person's general confidence in their capability to organize and carry out the actions necessary for achieving intended results (Locke, 1997). COR theory states that sustaining or acquiring psychological resources under stress correlates with an individual's adaptive capacity and confidence level (Hobfoll, 1989). As a core personal resource in the COR, self-efficacy enables individuals to cope with stress and maintain motivation across various life domains, including the workplace. In educational settings, teachers often encounter challenges such as classroom management difficulties, teaching setbacks, or communication issues with parents. More self-compassion is linked to a kinder and more understanding perspective toward oneself. This tends to coincide with lower self-criticism and lower perceived depletion of personal resources (Leung et al., 2024). This positive regulation of internal resources may help sustain individuals' general efficacy beliefs when facing work-related stressors (Jakobsson et al., 2025). For example, (Jang and Hong 2024) found that the self-compassion levels among early childhood teachers are significantly optimistically correlated with self-efficacy. Therefore, based on the above studies, this research further explores the relational link between early childhood teachers‘ self-compassion and self-efficacy, and proposes the hypothesis stated below:

H1: Self-compassion is positively linked to self-efficacy among early childhood teachers.

### 2.2 The mediating role of emotional exhaustion

Emotional exhaustion involves a deep sense of fatigue stemming from the ongoing overuse of psychological and emotional reserves. It commonly manifests as feelings of being emotionally depleted, physically and mentally worn out, and a loss of enthusiasm for work (Wright and Cropanzano, 1998). This construct, a central dimension of occupational burnout, captures the extent of internal emotional resource depletion from sustained work stress in individuals (Wu et al., 2023). COR theory proposes that individuals are prone to emotional exhaustion when they perceive a continuous loss or threat of loss to their key resources. These resources include time, emotional energy, and self-worth. This state occurs particularly when such losses cannot be effectively replenished (Hobfoll, 1989). In early childhood teaching, emotional exhaustion often shows as follows: facing young children's constant emotional needs, complex behavior guidance, frequent home-kindergarten communication, and non-teaching care duties, teachers gradually lose emotional responsiveness and teaching enthusiasm, and even avoid interactions or feel emotionally distant (Trauernicht et al., 2025). Self-compassion correlates negatively with emotional exhaustion (Chen et al., 2022; Tandler and Petersen, 2021). (Moè and Katz 2020) found teacher self-compassion to be significantly negatively correlated with job burnout, which included the dimension of emotional exhaustion. This negative relationship may reflect that self-compassion, as a self-regulatory resource, is associated with stronger psychological resource protection, which co-occurs with a stable negative relationship with emotional exhaustion.

A wealth of existing studies also corroborate the association between emotional exhaustion and self-efficacy (Chen, 2023; Costa et al., 2024). Self-efficacy is thought to relate to adequate psychological resources. In contrast, in a state of emotional exhaustion, an individual's emotional and cognitive resources tend to be heavily depleted, which co-occurs with difficulty in maintaining a positive belief in their teaching abilities (Li, 2023). In a survey of 527 early childhood teachers by (Yan et al. 2025), emotional exhaustion showed a significant negative correlation with self-efficacy. Consistent with this, (Öztürk et al. 2021) found emotional exhaustion to be significantly negatively correlated with the student engagement component of self-efficacy. In addition, studies suggest that emotional exhaustion frequently mediates the connection linking teachers‘ emotional experiences to their later professional outcomes. (Zhang et al. 2022) discovered emotional exhaustion to be a mediator of the relationship linking teachers' emotions to job satisfaction. Drawing on this theoretical framework and extant empirical cases, emotional exhaustion could function as a mediating factor connecting self-compassion with self-efficacy. To this end, we posit the following hypothesis:

H2: Among early childhood teachers, self-compassion and emotional exhaustion are inversely correlated.

H3: Emotional exhaustion correlates negatively with self-efficacy.

H4: Emotional exhaustion is a mediator of the link from self-compassion to self-efficacy.

### 2.3 The moderating role of core self-evaluation

Core self-evaluation denotes an individual's fundamental appraisal of their capabilities and value. It is a broad personality trait structure encompassing several fundamental psychological dimensions, including self-esteem, generalized efficacy, emotional stability, and perceived control (Judge et al., 1997). According to COR theory, resource depletion's influence on people's outcomes is not constant, but is instead influenced by the individual's existing resource reserves. The core self-evaluation, as a stable and comprehensive self-assessment system, reflects the profound psychological resource foundation that an individual relies on when facing challenges. The core self-evaluation may be associated with the way an individual manages the consumption of resources; however, it does not determine whether such consumption will occur (Hobfoll, 1989). Thus, we propose that core self-evaluation likely moderates the relationship between emotional exhaustion and self-efficacy. That is to say, teachers with higher core self-evaluation levels usually have greater self-worth and overall efficacy beliefs (Guo et al., 2019). Even when experiencing emotional exhaustion, these teachers may strategically utilize their psychological resources and actively respond to adverse situations (Palos, 2024). Empirical studies support this view. (Hong et al. 2025) studied 3,519 Chinese teachers. They found the relationship of emotional exhaustion to work meaning was moderated by core self-evaluation. Relevant studies also indicate that core self-evaluation correlates with lower emotional exhaustion (Arli et al., 2023) and higher occupational self-efficacy (Cao et al., 2025). Together, the evidence indicates that under emotional exhaustion, core self-evaluation differences may correspond to varying levels of self-efficacy. Thus, we put forward the hypothesis that:

H5: Core self-evaluation moderates the link connecting emotional exhaustion to self-efficacy.

**Figure 1:**
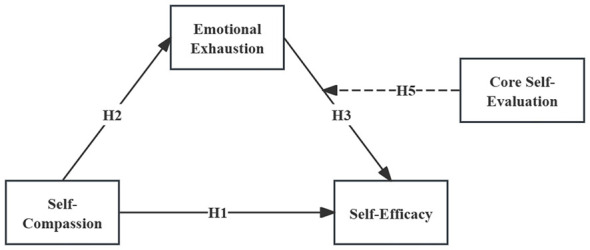
Research hypothesis model.

Guided by COR theory (Hobfoll, 1989), we regarded self-efficacy as the main research outcome. We constructed a moderated mediation model. The model conducts a systematic examination of emotional exhaustion's mediating role. Additionally, it tests how core self-evaluation modifies the connection linking emotional exhaustion to self-efficacy. Figure 1 presents the theoretical hypothesis model.

## 3 Methods

### 3.1 Sample and data collection

Data for this study were collected through the online survey platform Questionnaire Star (www.Sojump.com), and the proposed model was assessed. We collected data from November to December 2025. Prior to administering the questionnaire survey, we ensured that all participants understood the research aims. Moreover, every participant in this research engaged in the work voluntarily. They provided explicit consent for the protection of their private information and data privacy. We recruited early childhood teachers from China to ensure our sample was representative. Our final dataset comprised 584 completed questionnaires. Data from 32 participants were excluded for the following reason: those respondents who answered every question with repeated responses, indicating that they were not fully engaged during the survey (Schroeders et al., 2022). Therefore, the final analysis used 552 valid questionnaires. Table 1 displays the detailed demographic data of the people. 169 (30.6%) were male, and 383 (69.4%) were female. Regarding marital status, 383 participants were unmarried (69.44%). Regarding age distribution, 155 participants aged 20–30 (28.1%), 171 aged 31–40 (31.0%), 110 aged 41–50 (19.9%), and 116 aged 51 or above (21.0%). In terms of teaching experience, 194 participants had 6–10 years of experience (35.1%). Most participants worked in private kindergartens (406 participants, 73.5%). The most common education level was a bachelor's degree (239 participants, 43.3%). In this study, male participants made up 30.6% of the sample, which is substantially higher than what is typically observed among Chinese early childhood teachers. This over-representation may be related to the online and voluntary recruitment procedure, which may have introduced self-selection bias. Therefore, the gender distribution of this sample should not be interpreted as reflecting the actual gender structure of early childhood teachers in China, and caution is needed when applying these findings to broader populations.

**Table 1:** Descriptive statistics.

Participant demographics	Variable	Quantity	Percentage
Gender	Male	169	30.6%
	Female	383	69.4%
Marital status	Married	169	30.6%
	Unmarried	383	69.4%
Age	20–30 years	155	28.1%
	31–40 years	171	31.0%
	41–50 years	110	19.9%
	Above 51 years	116	21.0%
Years of teaching experience	1–5 years	154	27.9%
	6–10 years	194	35.1%
	11–15 years	102	18.5%
	More than 15 years	102	18.5%
Type of Kindergarten	Public	146	26.5%
	Private	406	73.5%
Education level	Associate degree	158	28.6%
	Bachelor's degree	239	43.3%
	Master's degree or above	155	28.1%

### 3.2 Measures

This study employed widely recognized and validated measurement scales, and the content and structure of the items were kept intact. Elevated scores indicate stronger variables assessed by the scales.

We assessed self-compassion using the Self-Compassion Scale developed by (Raes et al. 2011). The scale has 12 items, including 6 reverse-scored ones (e.g., When I feel inadequate in a certain aspect, I will try to remind myself: This feeling of incompetence is something that most people experience). It uses a 5-point Likert scale (Leung et al., 2024). Its Cronbach's α coefficient is 0.925.

Self-efficacy was assessed with the General Self-Efficacy Scale (Luszczynska et al., 2005). The scale includes of 10 items (e.g., “When I make the necessary effort, I am able to solve most problems”). A 4-point Likert scale is employed for scoring (Pan et al., 2022). Its Cronbach's α coefficient is 0.909.

Emotional exhaustion was assessed with the Emotional Exhaustion Scale (Maslach and Jackson, 1981). The scale has 9 items (e.g., Working directly with students causes me great stress). It uses a 7-point Likert scale (Wang et al., 2024). Its Cronbach's α coefficient is 0.915.

The Core Self-Evaluation Scale was used to assess core self-evaluation. The 10-item scale includes six reverse-scored items (e.g., “Most of my problems are situations I can manage”). The measure employs a 5-point Likert scale for scoring (Li, 2025). Its Cronbach's α coefficient is 0.911.

### 3.3 Data analysis

We first assessed the psychometric properties of the measurement instruments using the criteria commonly applied in PLS-SEM. This step served solely to evaluate convergent and discriminant validity of the constructs (i.e., factor loadings, composite reliability, HTMT, and Fornell-Larcker criterion). No structural PLS-SEM model was estimated.

We used the PROCESS macro 4.2 for SPSS to conduct mediation and moderation analyses. To examine the hypothesized mediation pathway, we applied Model 4. For testing moderation, we utilized Model 14. We employed the bootstrap method with 5,000 resamples to estimate the confidence interval of the indirect effect. 95% of the confidence intervals for the self-help method do not include zero, which indicates the presence of a significant indirect effect.

## 4 Results

### 4.1 Descriptive statistics and correlation analysis

Correlation analysis revealed that self-compassion was inversely correlated with emotional exhaustion (*r* = −0.364, *p* < 0.001) and was strongly positively linked to self-efficacy (*r* = 0.602, *p* < 0.001). The level of emotional exhaustion was inversely correlated with self-efficacy (*r* = −0.539, *p* < 0.001) (see Table 2).

**Table 2:** Correlation analysis.

Constructs	M±SD	Sk	Kur	Self-compassion	Emotional exhaustion	Core self-evaluation	Self-efficacy
Self-compassion	3.35 ± 0.79	−0.413	−0.647	1			
Emotional exhaustion	3.53 ± 1.04	0.153	−0.678	−0.364^***^	1		
Core self-evaluation	2.96 ± 0.76	−0.423	−0.961	0.202^***^	−0.629^***^	1	
Self-efficacy	2.88 ± 0.65	−0.828	0.059	0.602^***^	−0.539^***^	0.419^***^	1

^***^p < 0.001.

### 4.2 Common method bias

We adopted one approach to evaluate CMB. The results showed that a single factor did not account for the majority of the variance (Podsakoff et al., 2003). The largest single factor explained 33.292% of the total variance, remaining substantially under the critical threshold of 50% (Podsakoff et al., 2003). Therefore, following these results, we infer that the data did not show substantial CMB.

Subsequently, we implemented a confirmatory factor analysis (CFA) to further examine the structural validity of the measurement model. As shown in Table 3, the overall model fit was acceptable: χ^2^ = 749.437, df = 733, χ^2^/ df = 1.022, RMSEA = 0.006, SRMR = 0.032, GFI = 0.997, AGFI = 0.929, CFI = 0.997, IFI = 0.999, TLI = 0.998. These indices all met the standard requirements for structural equation modeling, indicating good overall model fit and acceptable convergent validity. In addition, we added a common method factor and conducted an unmeasured latent method factor (ULMC) model test. The results showed only minor changes in fit indices compared to the baseline model, indicating that common method bias did not substantially influence the findings (Marsh and Hocevar, 1985).

**Table 3:** CFA fit indices and comparison between baseline and ulmc models.

Model	*χ^2^/df*	RMSEA	SRMR	GFI	AGFI	CFI	IFI	TLI
Referenc*e* value	< 3	< 0.080	< 0.080	>0.800	>0.800	>0.800	>0.800	>0.800
Baseline model	1.022	0.006	0.032	0.939	0.929	0.997	0.999	0.998
Method factor model	1.010	0.004	0.031	0.940	0.930	0.999	0.999	0.999
Model fit difference		ΔRMSEA	ΔSRMR	ΔGFI	ΔAGFI	ΔCFI	ΔIFI	ΔTLI
		0.002	0.001	0.001	0.001	0.002	0.000	0.001
Evaluation criterion		< 0.05	< 0.05	< 0.01	< 0.01	< 0.01	< 0.01	< 0.01

CMIN chi-square value, DF: degree of freedom, RMSEA: root mean square error of approximation, SRMR: standardized root means square residual, GFI: goodness-of fit index, AGFI: adjusted goodness-of-fit index, CFI: comparative fit index, IFI: incremental fit-index, TLI: tucker-lewis-index.

### 4.3 Structural reliability and validity

Following the recommendations of (Hair Jr et al. 2021), we evaluated the reliability and validity of the scales using measurement model analysis. Item outer loadings and composite reliability were adopted as the key reliability indices. According to the guidelines, item outer loadings should exceed 0.708 (Hair Jr et al., 2021). As indicated in Table 4, all measurement items yielded satisfactory outer loadings, with all latent constructs showing high composite reliability. We then assessed validity by examining convergent and discriminant validity. Convergent validity is indicated by an average variance extracted (AVE) value above 0.50 (Hair Jr et al., 2021). Discriminant validity was evaluated through both the heterotrait–monotrait ratio of correlations (HTMT) and the Fornell-Larcker criterion. All HTMT values were below the suggested cutoff of 0.85 (see Table 5) (Hair Jr et al., 2021). For each pair of constructs, the correlations did not exceed the square root of their respective AVE (see Table 6), satisfying the Fornell-Larcker criterion (Hair Jr et al., 2021). These results, from both the HTMT and Fornell–Larcker approaches, indicate adequate discriminant validity for all constructs.

**Table 4:** Reliability and validity.

Constructs	Items	Loadings	CR	Cronbach's α	AVE
Self-compassion	Self-compassion 1	0.828	0.935	0.925	0.548
	Self-compassion 2	0.736			
	Self-compassion3	0.721			
	Self-compassion4	0.702			
	Self-compassion5	0.722			
	Self-compassion6	0.763			
	Self-compassion7	0.733			
	Self-compassion8	0.747			
	Self-Compassion9	0.753			
	Self-compassion10	0.722			
	Self-compassion11	0.708			
	Self-compassion12	0.736			
Emotional exhaustion	Emotional exhaustion1	0.784	0.930	0.915	0.595
	Emotional exhaustion2	0.788			
	Emotional exhaustion3	0.784			
	Emotional exhaustion4	0.755			
	Emotional exhaustion5	0.768			
	Emotional exhaustion6	0.784			
	Emotional exhaustion7	0.765			
	Emotional exhaustion8	0.768			
	Emotional exhaustion9	0.746			
Core self-evaluation	Core self-evaluation1	0.754	0.926	0.911	0.555
	Core Self-evaluation2	0.790			
	Core self-evaluation3	0.726			
	Core self-evaluation4	0.740			
	Core self-evaluation5	0.741			
	Core self-evaluation6	0.718			
	Core self-evaluation7	0.727			
	Core self-evaluation8	0.762			
	Core self-evaluation9	0.743			
	Core self-evaluation10	0.745			
Self-efficacy	Self-efficacy1	0.786	0.924	0.909	0.550
	Self-Efficacy2	0.708			
	Self-Efficacy3	0.718			
	Self-Efficacy4	0.776			
	Self-efficacy5	0.734			
	Self-efficacy6	0.743			
	Self-efficacy7	0.747			
	Self-efficacy8	0.739			
	Self-efficacy9	0.736			
	Self-efficacy10	0.725			

CR, composite reliability; AVE, average variance extracted.

**Table 5:** Discriminant validity(HTMT standard).

Constructs	Emotional exhaustion	Self-efficacy	Core self-evaluation	Self-compassion
Emotional exhaustion				
Self-efficacy	0.591			
Core self-evaluation	0.689	0.461		
Self-compassion	0.399	0.656	0.223	

For discriminant validity to be established, all values must be maintained under the cutoff of 0.85.

**Table 6:** Fornell-larcker criteria.

Constructs	Emotional exhaustion	Self-efficacy	Core self-evaluation	Self-compassion
Emotional exhaustion	***0.771***			
Self-efficacy	−0.542	***0.741***		
Core self-evaluation	−0.632	0.423	***0.745***	
Self-compassion	−0.369	0.605	0.205	***0.740***

The Fornell-Larcker criterion stipulates that for discriminant validity to hold, each construct's correlation with others must not exceed the square root of its own AVE (shown in bold italics on the diagonal).

### 4.4 Collinearity test

We assess the possible multicollinearity in the model by examining the variance inflation factor (VIF) values of all potential constructs (Hair Jr et al., 2021). All VIF values fell under the established critical threshold of 3.3. This confirms that multicollinearity does not present a significant concern for the model's results (Hair Jr et al., 2021). Table 7 confirms that multicollinearity does not alter the study's findings.

**Table 7:** VIF.

Constructs	Emotional exhaustion	Self-efficacy	Core self-evaluation	Self-compassion
Emotional exhaustion		1.856		
Self-efficacy				
Core self-evaluation		1.717		
Self-compassion	1.000	1.160		

Furthermore, to further whether gender differences might influence the main study variables, independent-samples t-tests were performed for self-compassion, emotional exhaustion, core self-evaluation, and self-efficacy. As shown in Table 8, none of the four core variables differed significantly by gender. In addition, gender was treated as a control variable in the main model. Together, these results suggest that gender was unlikely to substantially confound the hypothesized relationships in this study.

**Table 8:** *t*-test analysis by gender.

Constructs	Male (n = 169)		Female (*n* = 383)		*T*	*P*	Cohen's d	95%CI	
	M	SD	M	SD				LLCI	ULCI
Emotional exhaustion	−0.010	1.070	0.004	0.969	−0.150	0.198	−0.014	−0.195	0.167
Self-efficacy	−0.065	1.065	0.029	0.970	−1.020	0.108	−0.094	−0.275	0.087
Core self-evaluation	−0.013	0.998	0.057	1.002	−0.201	0.991	−0.019	−0.200	0.162
Self-compassion	−0.099	1.059	0.044	0.971	−1.549	0.198	−0.143	−0.324	0.038

### 4.5 Mediating role analysis

The results of the correlation analysis satisfy the necessary statistical conditions for proceeding with the mediation hypothesis testing. Based on this, we used the PROCESS macro (Model 4) in SPSS Statistics 26. The bias-corrected percentile Bootstrap method with 5,000 resamples was used. Focusing on early childhood teachers, the analysis considered how self-compassion relates to self-efficacy. The role of emotional exhaustion was tested to determine if it mediated the relationship. In the analysis, we incorporated demographic factors (e.g., gender, age, marital status) as covariates, and all variables were standardized. The regression results are summarized in Table 9.

**Table 9:** Regression results for the mediation model.

Outcome variable	Predictor variable	β	*SE*	*T*	Bootstrap 95% CI		*R^2^*	*F*
					LLCI	ULCI		
Emotional exhaustion	Self-compassion	−0.365	0.040	−9.118^***^	−0.444	−0.286	0.134	12.018
	Gender	0.031	0.087	0.782	−0.103	0.239		
	Marital status	−0.005	0.087	−0.122	−0.181	0.160		
	Age	0.011	0.036	0.264	−0.062	0.081		
	Years of teaching experience	−0.012	0.038	−0.306	−0.086	0.063		
	Type of kindergarten	−0.015	0.091	−0.374	−0.212	0.144		
	Education level	0.011	0.033	0.273	−0.090	0.119		
Self-efficacy	Self-compassion	0.467	0.033	14.105^***^	0.402	0.532	0.486	64.306
	Emotional exhaustion	−0.369	0.033	−11.160^***^	−0.434	−0.304		
	Gender	0.016	0.067	0.515	−0.097	0.166		
	Marital status	−0.066	0.067	−2.136^*^	−0.274	−0.011		
	Age	−0.008	0.028	−0.248	−0.062	0.048		
	Years of teaching experience	0.041	0.029	1.331	−0.018	0.096		
	Type of kindergarten	−0.016	0.070	−0.515	−0.173	0.101		
	Education level	−0.013	0.041	−0.407	−0.097	0.064		

N = 552; ^*^p < 0.05; ^**^p < 0.01; ^***^p < 0.001.

Self-compassion was negatively associated with emotional exhaustion (β = −0.365, *p* < 0.001). Self-efficacy showed a positive correlation with self-compassion, a relationship that was statistically significant (β = 0.467, *p* < 0.001). Furthermore, higher emotional exhaustion is strongly linked to lower self-efficacy (β = −0.369, *p* < 0.001). The model had strong explanatory power, with an *R*^2^ value of 0.486 for self-efficacy, indicating a good model fit. The *R*^2^ value for emotional exhaustion was 0.134. Although the path coefficient was significant (β = −0.365, *p* < 0.001), the low coefficient of determination indicates that emotional exhaustion is also influenced by many factors not included in this model (such as workload, administrative support, and children's behavioral problems, etc.). Additionally, regression analysis showed that one covariate significantly correlated with self-efficacy. Overall, the above results provide statistical support for further testing of the mediation role of emotional exhaustion linking self-compassion to self-efficacy in early childhood teachers.

As shown in Table 10, the overall influence of self-compassion on self-efficacy was 0.602 (95% bootstrap CI [0.535, 0.669]). Its direct effect was 0.467 (95% bootstrap CI [0.402, 0.532]). The indirect effect value was 0.135 (95% bootstrap CI [0.094, 0.175]).

**Table 10:** Mediation analysis.

Effect	Path	β	Bootstrap 95% CI	
			LLCI	ULCI
Total effect	Self-Compassion → Self-Efficacy	0.602	0.535	0.669
Direct effect	Self-Compassion → Self-Efficacy	0.467	0.402	0.532
Indirect effect	Self-Compassion → Emotional Exhaustion → Self-Efficacy	0.135	0.094	0.175

*N* = 552.

### 4.6 Moderation analysis

**Figure 2:**
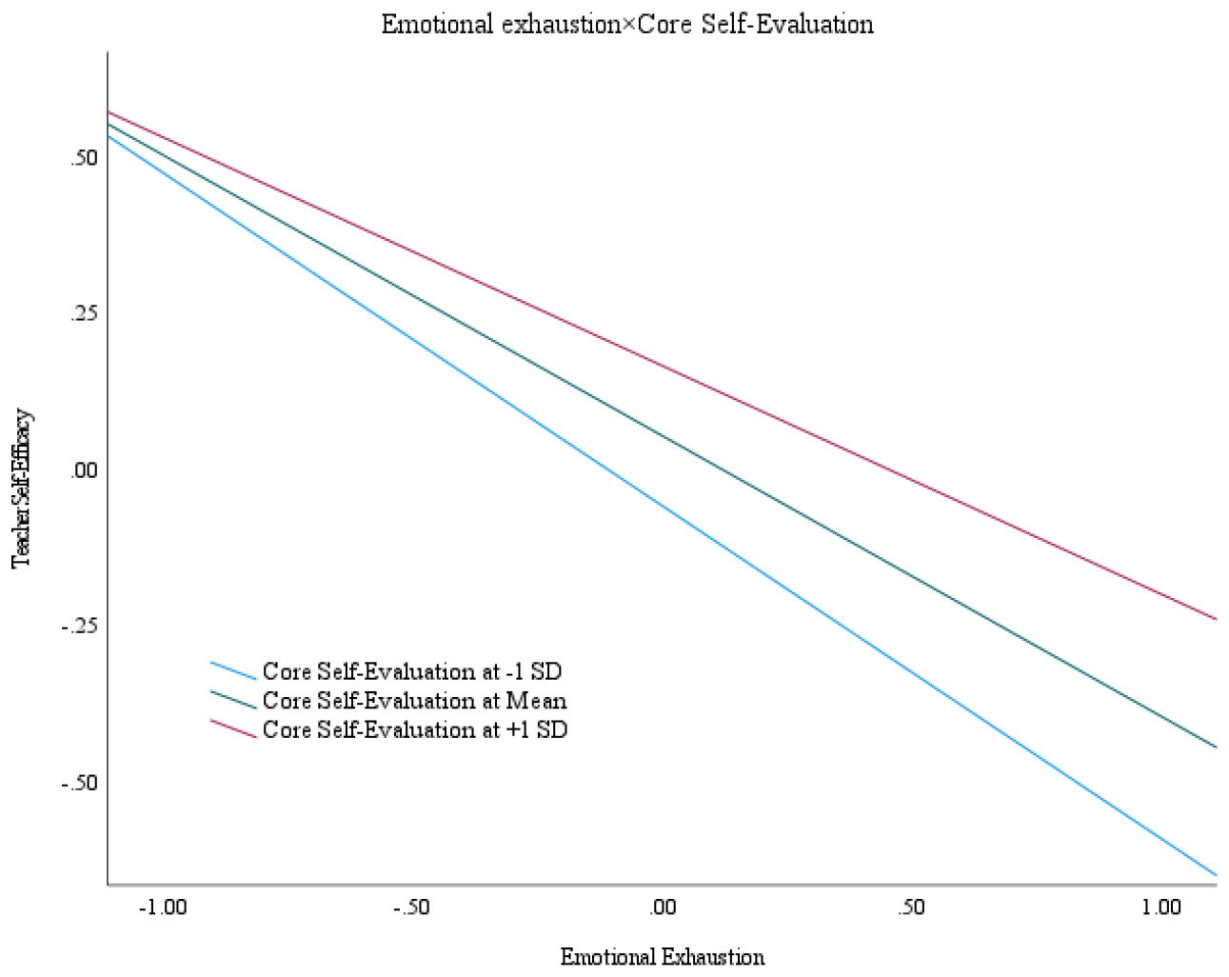
Core self-evaluation moderates the connection between emotional exhaustion and self-efficacy.

We used Model 14 in the SPSS PROCESS macro to test the hypothesized moderated mediation model. As presented in Table 11. Core self-evaluation as a significant moderator connecting emotional exhaustion to self-efficacy (β = 0.083, *T* = 2.913). Figure 2 further reveals that the negative link from emotional exhaustion to self-efficacy is more pronounced when core self-evaluation is low.

**Table 11:** Moderation analysis.

Outcome variable	Predictor variable	β	*SE*	*T*	Bootstrap 95% CI		*R^2^*	Δ*R^2^*
					LLCI	ULCI		
Emotional exhaustion	Self-compassion	−0.365	0.0467	−9.118^***^	−0.456	−0.273	0.134	
Self-efficacy	Emotional exhaustion	−0.262	0.050	−6.406^***^	−0.365	−0.163	0.503	
Self-efficacy	Core self-evaluation	0.137	0.044	3.470^**^	0.051	0.220		
Self-efficacy	Self-compassion	0.473	0.043	14.558^***^	0.390	0.557		
Self-Efficacy	Emotional exhaustion × core self-evaluation	0.083	0.028	2.913^**^	0.027	0.138		0.008

*N* = 552; ^**^p < 0.01; ^***^p < 0.001.

## 5 Discussion

This work conducted a cross-sectional survey of 552 Chinese early childhood teachers, based on the COR theory. It explored the link from self-compassion to self-efficacy, together with the mediation of emotional exhaustion and the moderation of core self-evaluation. All proposed hypotheses were supported by the data. The discussion now turns to a detailed examination of these findings.

Greater self-compassion and higher levels of self-efficacy were positively correlated among early childhood teachers, supporting H1. Our result aligns with the conclusions drawn by (Jang and Hong 2024). According to COR theory, how resources are conserved relates to adaptability and confidence levels. Continuous depletion of psychological resources is usually associated with a decline in an individual's ability (Hobfoll, 1989). Early childhood education, as a profession, is characterized by the need for a significant amount of emotional investment and continuous emotional labor. Teachers often face challenges such as managing complex classroom dynamics, promoting communication between home and school, and responding to the unique individual needs of children (Li et al., 2020). Under such conditions, high levels of self-compassion are often accompanied by more flexible emotion regulation. This pattern is also related to a lower sense of internal resource depletion associated with self-criticism (Chan and Halim, 2025). Therefore, self-compassion can be regarded as a crucial personal trait. This trait may coincide with a balance of psychological resources and is associated with greater levels of self-efficacy.

Emotional exhaustion mediates the link connecting self-compassion to self-efficacy, verifying H2, H3, and H4. This mediating pattern aligns with COR theory (Hobfoll, 1989), stating that the dynamic balance of psychological resources is related to career adaptation and perceived ability. Emotional exhaustion, as a core indicator of resource depletion, may function as a linking mechanism from self-compassion to occupational efficacy beliefs (Hobfoll et al., 2018). In the study, self-compassion demonstrated a significant inverse relationship with emotional exhaustion, consistent with prior findings that self-kindness and mindful awareness co-occur with lower emotional exhaustion in teachers (Chen et al., 2022; O'Hara-Gregan, 2024). In addition, an inverse relationship exists linking emotional exhaustion to self-efficacy. When experiencing emotional exhaustion, teachers tend to have fewer available psychological resources, which co-occurs with lower professional self-confidence (Yan et al., 2025). This further clarifies the negative correlation between psychological resource depletion and self-efficacy (Xu and Jia, 2022). Based on these results, interventions aimed at cultivating self-compassion and reducing emotional exhaustion may be associated with maintaining and enhancing self-efficacy among early childhood teachers.

Core self-evaluation serves as a moderator linking emotional exhaustion to self-efficacy, verifying H5. This study found that individuals with higher core self-evaluation scores exhibited stronger self-esteem and emotional stability, traits that were associated with greater psychological resource reserves and with a more stable sense of self-efficacy (Cao et al., 2025; Saei et al., 2024). From the perspective of COR theory (Hobfoll, 1989), teachers with higher core self-evaluation likely possess a stronger baseline of psychological resources and demonstrate a more proactive attitude toward resource protection. In early childhood education, which demands high emotional labor and continuous emotional investment, resource loss is a common experience (Purper et al., 2023). When experiencing emotional exhaustion, teachers who have higher core self-evaluation tend to form a more positive and comprehensive perception of their own teaching abilities. For these teachers, emotional exhaustion is more often viewed as a temporary phase in their professional work rather than as a sign of incompetence (Peláez-Fernández et al., 2022). This kind of cognition is associated with a positive belief in the professional capabilities of teachers. This factor may alleviate the negative link from emotional exhaustion to self-efficacy. The results indicate that the core self-evaluation can serve as a potential intervention point to alleviate the negative relationship from emotional exhaustion to self-efficacy in early childhood teachers.

### 5.1 Implications

Guided by COR theory, we constructed and validated a moderated mediation model. We developed this model to offer a nuanced examination of the interplay between self-compassion and self-efficacy, focusing specifically on early childhood educators.

#### 5.1.1 Theoretical implications

This work offers more empirical evidence for the use of COR theory in the psychological functions of early childhood teachers. It regards self-compassion as a fundamental and intrinsic psychological resource for these teachers. The study also views emotional exhaustion as a central sign of psychological resource depletion. Furthermore, the work investigates the mediating pathway linking self-compassion to self-efficacy. It confirms that emotional exhaustion serves as a mediator between these two constructs. These findings support the core principles of COR theory among early childhood teachers. They offer an empirical basis for applying the theory to future research on preschool teachers' professional wellbeing.

Secondly, this study provides further explanation of individual differences in the process of psychological resource functioning by verifying the core self-evaluation's moderating role. According to the work, the extent of the extent of the correlation of emotional exhaustion and self-efficacy may vary depending on an individual's level of core self-evaluation. Specifically, for teachers who have higher core self-evaluation, the negative link connecting emotional exhaustion to self-efficacy appeared less pronounced. Conversely, this association was relatively stronger among teachers with lower core self-evaluation. In addition, between marital status and self-efficacy, this work detected a statistically significant association. This finding suggests that when examining the composition and flow of teachers' psychological resources, external social support systems, such as those from intimate relationships, may also serve as important contextual resource variables and have a certain connection with self-efficacy.

#### 5.1.2 Practical implications

Emotional exhaustion acts as a mediator in the relational connection of self-compassion and self-efficacy among early childhood teachers. This finding opens up new directions for mental health interventions. For early childhood teachers, self-compassion training may help protect psychological resources. For example, teachers can briefly note one or two small achievements during their teaching day, for example, successfully guiding children to resolve a conflict. Adopting a compassionate and accepting attitude toward one's own teaching may also reduce internal strain associated with self-criticism. When experiencing emotional exhaustion, simple emotion regulation exercises, such as brief mindful breathing, may help alleviate immediate resource depletion. Core self-evaluation moderates the association of emotional exhaustion with self-efficacy. Teachers may strengthen their core self-evaluation by reflecting on their own teaching strengths and engaging in peer experience exchange, which may help stabilize a positive perception of their teaching abilities. For teachers who experience emotional exhaustion due to occupational stress, distinguishing between normal emotional demands of the profession and perceived personal weaknesses may be useful, as this cognitive distinction is associated with a weaker negative connection linking emotional exhaustion to self-efficacy.

This study reveals a negative link from emotional exhaustion to self-efficacy, highlighting the importance of a supportive organizational environment for protecting teachers' psychological resources. For early childhood administrators and institutions, such an environment can be fostered by integrating teacher mental health into daily operations. Workload optimization is recommended, for example, avoiding frequent overtime and excessive emotional labor arrangements. Clear boundaries for teacher working hours can be set, such as not replying to non-urgent parent messages after 8 p.m. on weekdays. When scheduling classes, the intensity of emotional labor may be taken into account, for example, avoiding assigning the same teacher to high-demand classes consecutively. Low-cost, low-pressure support mechanisms can be implemented, such as monthly 15–20 min peer sharing sessions on emotion management led by senior teachers with no need for external speakers, and monthly 5–10 min one-on-one non-judgmental conversations between managers and each teacher to understand work concerns.

### 5.2 Limitations and future research

This work offers initial evidence on how self-compassion relates to self-efficacy among early childhood teachers. It explores emotional exhaustion as a mediator and core self-evaluation as a moderator in this relationship. But the current research has some limitations and points out the potential directions for future studies. In this cross-sectional study, data were collected at one time point, and the relationships among the variables were examined. This cannot capture the dynamic development process and long-term relationship characteristics among the variables. Future research could employ longitudinal designs. This enables a systematic study of the long-term dynamic relationships between variables. The use of cross-lagged panel analysis and other statistical methods is helpful in clarifying the causal relationships between variables. This approach would strengthen the causal inference and stability of the research conclusions. Furthermore, all research data in this study were collected through self-reporting. This design may be influenced by social expectation bias, which might potentially undermine the objectivity and accuracy of the research conclusions. A single data collection method may also lead to an overestimation of the correlations between variables. Future research could integrate multiple assessment methods. These methods may include interviews and behavioral observations. Adopting this multi-method approach will enhance the objectivity and credibility of the collected data. Third, in addition to emotional exhaustion and core self-evaluation, there may be other unrecognized potential mediators and moderators (e.g., leadership care and peer support) that have not been incorporated into this model. Future studies could explore a broader range of potential factors. Fourth, this study used a general self-efficacy scale, which assesses cross-domain personal resources rather than teacher-specific efficacy. This may constrain the generalizability of the present observations to the teaching context. Future research should replicate the model using a domain-specific instrument, such as the Teacher Self-Efficacy Scale. Finally, this study's male participation rate (30.6%) greatly exceeds the actual male teacher ratio in the Chinese early childhood education workforce. This over-representation may restrict the applicability of our results to the broader population of early childhood teachers. Future research should adopt stratified sampling to achieve a more representative gender distribution.

It is worth noting that core self-evaluation and emotional exhaustion were strongly correlated (*r* = −0.629). Whereas emotional exhaustion is a transient state of resource depletion, core self-evaluation is a stable personality trait that includes self-esteem, generalized self-efficacy, emotional stability, and perceived control. This strong correlation may suggest that people who are low in core self-evaluation are more susceptible to emotional exhaustion, or that prolonged exhaustion tends to co-occur with lower levels of core self-evaluation. Although the two constructs may be conceptually distinct, their empirical overlap indicates shared variance. Future longitudinal research could examine the causal ordering and discriminant validity of these two constructs.

## 6 Conclusion

This work examines how self-compassion relates to self-efficacy among early childhood educators, while further testing emotional exhaustion as a mediator in their association and core self-evaluation as a moderator in the association of emotional exhaustion with self-efficacy. We conducted an empirical test of the four hypotheses using self-reported data from 552 early childhood teachers. The study results demonstrate that self-compassion is significantly and positively associated with self-efficacy. The findings also confirm that emotional exhaustion acts as a significant mediator in this link. Additionally, core self-evaluation provides a significant positive moderating function for the relational link from emotional exhaustion to self-efficacy. We found each of these hypotheses to be corroborated. This work helps to understand the association of self-compassion of preschool teachers with self-efficacy as teachers. We also clarify the potential mechanisms of this relationship.

## Data Availability

The raw data supporting the conclusions of this article will be made available by the authors, without undue reservation.
